# Interference of Interleukin-1*β* Mediated by Lentivirus Promotes Functional Recovery of Spinal Cord Contusion Injury in Rats via the PI3K/AKT1 Signaling Pathway

**DOI:** 10.1155/2022/6285099

**Published:** 2022-10-03

**Authors:** Jun-Feng Cao, Xi Hu, Li Xiong, Mei Wu, Xingyu Yang, Chaochao Wang, Shengyan Chen, Hengxiang Xu, Huanyu Chen, Xuntai Ma, Yongjie Mi, Xiao Zhang

**Affiliations:** ^1^Clinical Medical College of Chengdu Medical College, Chengdu, China; ^2^The First Affiliated Hospital of Chengdu Medical College, Chengdu, China; ^3^Taikang Tongji Wuhan Hospital, Wuhan, China; ^4^Basic Medical College of Chengdu Medical College, Chengdu, China; ^5^National Demonstration Center for Experimental Clinical Medicine Education of Chengdu Medical College, Chengdu, China

## Abstract

**Purpose:**

Inflammation and apoptosis after spinal cord contusion (SCC) are important causes of irreversible spinal cord injury. Interleukin-1*β* (IL-1*β*) is a key inflammatory factor that promotes the aggravation of spinal cord contusion. However, the specific role and regulatory mechanism of IL-1*β* in spinal cord contusion is still unclear. Therefore, this study applied bioinformatics to analyze and mine potential gene targets interlinked with IL-1*β*, animal experiments and lentiviral interference technology were used to explore whether IL-1*β* affected the recovery of motor function in spinal cord contusion by interfering with PI3K/AKT1 signaling pathway.

**Method:**

This study used bioinformatics to screen and analyze gene targets related to IL-1*β*. The rat SCC animal model was established by the Allen method, and the Basso Beattie Bresnahan (BBB) score was used to evaluate the motor function of the spinal cord-injured rats. Immunohistochemistry and immunofluorescence were used to localize the expression of IL-1*β* and AKT1 proteins in spinal cord tissue. Quantitative polymerase chain reaction and Western blot were used to detect the gene and protein expressions of IL-1*β*, PI3K, and AKT1. RNAi technology was used to construct lentivirus to inhibit the expression of IL-1*β*, lentiviral interference with IL-1*β* was used to investigate the effect of IL-1*β* and AKT1 on the function of spinal cord contusion and the relationship among IL-1*β*, AKT1, and downstream signaling pathways.

**Results:**

Bioinformatics analysis suggested a close relationship between IL-1*β* and AKT1. Animal experiments have confirmed that IL-1*β* is closely related to the functional recovery of spinal cord contusion. Firstly, from the phenomenological level, the BBB score decreased after SCC, IL-1*β* and AKT1 were located in the cytoplasm of neurons in the anterior horn of the spinal cord, and the expression levels of IL-1*β* gene and protein in the experimental group were higher than those in the sham operation group. At the same time, the expression of AKT1 gene decreased, the results suggested that the increase of IL-1*β* affected the functional recovery of spinal cord contusion. Secondly, from the functional level, after inhibiting the expression of IL-1*β* with a lentivirus-mediated method, the BBB score was significantly increased, and the motor function of the spinal cord was improved. Thirdly, from the mechanistic level, bioinformatics analysis revealed the relationship between IL-1*β* and AKT1. In addition, the experiment further verified that in the PI3K/AKT1 signaling pathway, inhibition of IL-1*β* expression upregulated AKT1 gene expression, but PI3K expression was unchanged.

**Conclusion:**

Inhibition of IL-1*β* promotes recovery of motor function after spinal cord injury in rats through upregulation of AKT1 expression in the PI3K/AKT1 signaling pathway. Bioinformatics analysis suggested that IL-1*β* may affect apoptosis and regeneration by inhibiting the expression of AKT1 in the PI3K/AKT1 signaling pathway to regulate the downstream FOXO, mTOR, and GSK3 signaling pathways; thereby hindering the recovery of motor function in rats after spinal cord contusion. It provided a new perspective for clinical treatment of spinal cord contusion in the future.

## 1. Introduction

Spinal cord contusion (SCC) is a severe trauma of the central nervous system due to external forces. SCC can cause sensorimotor dysfunction below the level of injury and lead to disability and death. Complications caused by primary spinal cord tissue injury include inflammation, edema, and apoptosis among other conditions [1]. The complicated pathophysiologic process of SCC involves numerous factors such as inflammatory, growth, and signaling pathway factors. Many studies have shown that inflammatory factors interfere with cell survival, regeneration, and apoptosis [2, 3]. However, it has not been clearly demonstrated whether inflammatory factors are involved in the pathophysiological process of SCC.

Interleukin-1*β* (IL-1*β*) is one of the most extensively studied proinflammatory cytokines. IL-1*β* is produced by microglia, astrocytes, and neurons and has many functions after spinal cord contusion (SCC) [4]. IL-1*β* is able to induce a number of cellular reactions such as changes in intracellular calcium concentration [5]. It has been also demonstrated to have strong effects on astrocytes by promoting their activation, as well as proliferation and production of neurotoxic mediators [2]. Interestingly, healthy neurons have been found to be injured when exposed to IL-1*β*, and IL-1*β* can also aggravate neurodegeneration that leads to cell death in experimental models of central nervous system (CNS) trauma [6, 7]. Moreover, IL-1*β* has been shown to exacerbate lesions and activate microglia in animal models of SCC, thereby enlarging the lesion area. And this effect can be attenuated by exposure to IL-1 receptor antagonist (IL-1ra) [8]. Meanwhile, study found that axonal plasticity was improved in IL-1 gene deficient mice [9, 10]. It is known that IL-1*β* is important for tissue repair following SCC, but the mechanism of how IL-1*β* impacts the restoration of locomotor function in SCC has not yet been definitively reported.

Serine-threonine protein kinase is encoded by the AKT1 gene, which acts as a downstream target of phosphatidylinositol 3-kinase (PI3K) and is thought to be central to the PI3K/AKT signaling pathway [11]. AKT1 is activated by platelet-derived growth factors and it is a key mediator of growth factor-induced neuronal survival in the developing nervous system. By activating AKT1, survival factors can inhibit apoptosis in a transcription-independent manner [12]. Moreover, AKT1 inhibits neuronal apoptosis, thus affecting the recovery of motor function [13]. However, whether AKT1 is affected by IL-1*β* to restrain the restoration of motor function in SCC has not been reported, and there are no studies to elucidate the relationship between IL-1*β* and AKT1 in SCC.

We took the inflammatory factor IL-1*β* as the starting point to explore its mechanism of action in spinal cord contusion. We used bioinformatics to analyze the potential related gene targets of IL-1*β* and their possible involvement in pathological processes. However, bioinformatics analysis could not reveal the relationship between IL-1*β* and potentially relevant gene targets. Therefore, we applied experimental validation to explore the mechanism of action of inflammatory factors in spinal cord contusion. Firstly, we observed the expression and localization of IL-1*β* in the spinal cord contusion rat model. Secondly, IL-1*β* expression was inhibited by lentivirus-mediated RNAi method to explore whether IL-1*β* affects the recovery of spinal cord motor function. Finally, we explored whether IL-1*β* actually interferes with the PI3K/AKT1 signaling pathway.

Our results showed that IL-1*β* impeded the recovery of motor function after SCC in rats by disrupting AKT1 expression in the PI3K/AKT1 signaling pathway. And bioinformatics analysis suggested that AKT1 may affect apoptosis, regeneration, and recovery of motor function after SCC in rats by regulating the downstream FOXO, mTOR, and GSK3 signaling pathways.

## 2. Materials and Methods

### 2.1. Bioinformatics Analysis

#### 2.1.1. IL-1*β*-Related Potential-Associated Protein Target Analysis and Network Map Construction

To elucidate the interactions between potential protein targets, we screened potential key protein targets for the regulation of inflammatory responses by protein-protein interactions. The screening results provide a solid foundation for exploring potential downstream signaling pathways of IL-1*β*. String database (http://string-db.org/) covers the majority of known human protein-protein interaction information. All potential therapeutic protein targets of IL-1*β* were imported into Cytoscape 3.7.1 and analyzed by string plug-in [14], the protein type was defined as “Rattus norvegicus”, the relevant information on protein interactions was obtained which was saved as TSV format file. Afterwards, the network topology parameters were analyzed by Cytoscape 3.7.1, and the hub protein targets were screened according to the criteria of node degree value and median centrality value greater than the average value.

#### 2.1.2. The Gene Target Enrichment Analysis

In this study, GO and KEGG enrichment analysis were used to integrate and analyze the information of potential key protein targets of IL-1*β* in molecular interaction network, genome, and related signaling pathways. Therefore, we could analyze the regulatory role of IL-1*β* in spinal cord contusion more deeply and comprehensively. Interacting gene targets was used in the DAVID database (https://david.ncifcrf.gov/) for gene ontology (GO) functional annotation and Kyoto Encyclopedia of Genes and Genomes (KEGG) enrichment analysis. Molecular functions (MF), cellular components (CC), and biological processes (BP) of protein targets or gene targets were enriched by GO. The main signaling pathways and biological processes of IL-1*β* in spinal cord contusion were analyzed by enriching the KEGG pathway for the signaling pathways involved in the target and performing gene target screening under the condition of *p* < 0.05. The OmicShare tools platform (https://www.omicshare.com/tools/Home/Soft/roc) was used to visualize the results of GO enrichment and KEGG enrichment.

### 2.2. Animals and Groupings

All experimental procedures were performed in accord with the National Institutes of Health Guide for the Care and Use of Laboratory Animals (2011) and were approved by the Institute of Translational Neuroscience Center of Sichuan University, Chengdu, China, and by the institutional ethics and licensing committee of Chengdu Medical College, Chengdu, China.

A total of 144 female Sprague-Dawley (SD) rats (each weighing 200 ± 20 *g*) were purchased from the Animal Experimental Center, Medical Sciences Department of Sichuan University. The animals were individually housed, in quiet room, under 12/12 hours light/dark condition with free access to water and food. Animals were randomly divided into four groups: the sham; the spinal cord contusion injury (SCC); the vector; and the lentivirus mediated siRNA interference (IL-1*β*-RNAi-LV). Spinal cord contusion injury group was further divided into the following post-injury subgroups with six animals each: 6 hours (6 h), 12 hours (12 h,) 1 day (1d), 3 days (3d), and 28 days (28d). The lentivirus-mediated siRNA interference group consisted of the following subgroups of six animals each: 3 days group (3d), 7 days group (7d) and 28 days group (28d).

### 2.3. Preparation of Lentiviral Vector

Information on siRNA fragment with the highest interference efficiency was provided by GeneCopoeia, Guangzhou, China. We then constructed the IL-1*β* lentiviral expression vector, which expresses a gene encoding with red fluorescent protein (RFP). Thereafter, IL-1*β*-lentiviral expression vectors (5 *μ*g) and viral packaging vectors (1 *μ*L) were cotransfected into packaging cell line (293 T) to produce lentiviral particles (IL-1*β*-RNAi-LV). The viral supernatant was harvested at 48 hours post-transfection and filtered through a 0.45 *μ*m cellulose acetate filter. Then 5 mL of cell supernatant containing lentivirus was centrifuged (3500 × *g*, 25 minutes). The precipitate was redissolved in 500 *μ*L phosphate-buffered saline. Finally, the lentivirus was frozen at −80°C. The negative plasmid was also packaged and used as a negative control, designated as NC-LV, which theoretically had no effect on any gene.

### 2.4. Spinal Cord Contusion Animal Model

The rats were intraperitoneally injected with 2% sodium pentobarbital (0.1 mL/kg) and placed in a prone position on the operating table [15, 16]. Assess the depth of anesthesia by judging the toes of rats. Under aseptic conditions, an incision was made along the dorsal midline to expose the soft tissues and muscles, the yellow ligaments overlying the spinal end plate at T11, and finally the spinal cord. A microsyringe with 10 *μ*L virus mixture was injected 4 mm deep, 5 *μ*L each caudally and cranially to the spinal cord. At 48 hours post injection, bilateral laminectomy was performed at T11. To induce SCC, a 10 g weight was dropped from a height of 30 mm onto the exposed cord at T11. Urinary bladders of rats were manually expressed twice a day for one week until normal urination was restored. Rats in the sham group received laminectomy without cord injury. The animals were placed in a warm environment (33-35°C) 24 hours following the procedures. During this period, they were observed for infection (blood in urine, foul odor, and whitish color).

### 2.5. Locomotor Function Assessment

Restoration of hind limb function was assessed by the Basso Beattie Bresnahan (BBB) locomotor scale on days 1, 3, 5, 7, 14, and 28 postinjury. The 28-day group animals were placed in a 2-meter square cardboard box and observed by three people who were blinded to treatment of the rats [15]. The animals were observed according to the standard BBB grading standards and the recovery of motor function in the hind limbs was recorded. All observations were performed simultaneously. Scoring criteria were as follows: 0-7, joint activity; 8-13, gait and coordination function; 14-21, claw movement. Maximum score was 21 and hind limb paralysis was scored as 0.

### 2.6. Quantitative Polymerase Chain Reaction Analysis (qPCR)

Rats were intraperitoneally injected with 2% sodium pentobarbital (0.1 mL/kg) and perfused with 4% paraformaldehyde solution for 30 minutes. Damaged spinal cord tissues were carefully dissected and pretreated with the total RNA isolation reagent SuperfecTRI (Shanghai Pufei Biotechnology, Shanghai, China) and then centrifuged at 33500 g for 10 minutes before homogenizing. Total mRNA was extracted per the manufacturer's protocol and reverse transcription to cDNA was performed with the RevertAid First Strand cDNA Synthesis Kit (Thermo Scientific, Waltham, MA, USA). The kit was used for amplification, which included 160 *μ*L TE, 40 *μ*L cDNA, 12.5 *μ*L 2x PCR Mix, 0.6 *μ*L forward primer, 0.6 *μ*L reverse primer, 0.6 *μ*L TaqMan probe, and 7.7 *μ*L water. The PCR mixture underwent 45 cycles of 95°C for 2 minutes, 95°C for 15 seconds, 52°C for 20 seconds, and 60°C for 40 seconds to gain PCR amplification products. During the process, the *Δ*CT value was recorded, the relative content based on 2 − ΔΔCT value was calculated and inspected. Relative expressions were calculated with normalization to *β*-Actin values. Design and synthesis of primers (Table 1) and fluorescent probes were made. Gene sequences were obtained from GenBank (http://www.ncbi.nlm.nih.gov/gene; National Center for Biotechnology Information, U.S. National Library of Medicine, Bethesda, MD, USA) and primers were designed using Primer Premier 5 (Premier Biosoft International, Palo Alto, CA, USA). TaqMan fluorescent probes were constructed based on Applied Biosystems 7300 Real-Time Quantitative PCR System (Thermo Fisher Scientific) filter wavelength, FAM was selected as the fluorescent reporting group, and TAMRA was chosen for the quenching group. The primers and probes were synthesized by Sangon Biotech (Shanghai, China).

### 2.7. Western Blotting

Western blotting was used to determine the changes in IL-1*β* protein in the spinal cord in response to different treatments. The spinal cords at the lesion sites were harvested at different time points postinjury. The rats were deeply anesthetized with 2% sodium pentobarbital (0.1 mL/kg), and the spinal cords were dissected immediately. The tissues were homogenized on ice in 400 *μ*L of RIPA buffer (Thermo Fisher, Shanghai, China, #89900), containing a cocktail of phosphatase and protease, then centrifuged at 12000 × *g* for 30 minutes. Protein concentration of each sample was assayed with BCA reagent (Sigma, St. Louis, MO, USA, #GHS09). A 20 *μ*L aliquot of the samples was loaded and electrophoresed on 8% and 12% SDS polyacrylamide gel for 1.5 hours at 120 volts. Proteins were transferred from the gel to a polyvinylidene difluoride membrane. Then the membrane was blocked with 5% nonfat dry milk for 120 minutes. Primary antibody of *β*-Actin (1 : 100, mouse, #TA09, ZSGB-BIO, Beijing, China) and anti-IL-1*β* (1 : 100, rabbit, # AB234437, Abcam, Cambridge, MA, USA)were separately incubated with two target bands overnight at 4°C after the membranes were rinsed thrice in phosphate-buffered saline containing 0.05% Tween-20. The membrane was incubated for 2 hours with HRP-conjugated goat antirabbit IgG (1 : 2,000; ZSGB-BIO, #SP9000, Beijing, China) or goat antirabbit IgG (1 : 5,000; ZSGB-BIO, #SP9000, Beijing, China) for 2 hours at room temperature. Finally, the membranes were rinsed thrice in buffer and the immune complexes were quantified using the AlphaImager 2000 (Alpha Innotech, San Leandro, CA, USA) with Western Lightning Plus-enhanced chemiluminescence substrate (Perkin Elmer, Waltham, MA, USA). Protein densitometry analysis was performed by Quantity One 1-D analysis software (Bio-Rad, Hercules, CA, USA).

### 2.8. Immunohistochemistry

Rabbit IL-1*β* polyclonal antibody (1 : 100, rabbit, # AB234437, Abcam, Cambridge, MA, USA) was used as the primary antibody, while goat antirabbit IgG (1 : 100; Jackson Immunoresearch, # AB6788, West Grove, PA, USA) was used as the second antibody, and DAB chromogenic liquid was applied for detection of positive expression. The epicenters of injured cord tissues were embedded in methylmethacrylate plastic after serial dehydration with a graded ethanol series to xylene. The immune-positive cells were identified using an optical microscope. Five immunohistochemical slices were taken from each tissue. The ventral and dorsal horns of the spinal cord could be separated based on the vertical straight line drawn between central tube and dorsal medial sulcus of the spinal cord. Immune-positive cells with nuclei in the ventral horn were counted under a 10×-40× stereo microscope (Motic, Carlsbad, CA, USA).

### 2.9. Immunofluorescence

Primary antibodies used in this study contained rabbit IL-1*β* polyclonal antibody (1 : 100, rabbit, # AB234437, Abcam, Cambridge, MA, USA), rabbit AKT1 polyclonal antibody (1 : 150; Abcam, #AB179463), and NeuN (1 : 50, ZSGB-BIO, #ZM0352). Damaged spinal cord tissues were dehydrated, fixed, and embedded in paraffin, then cut into 5 *μ*m thick slices. The epicenter of injured spinal cord tissues were incubated in blocking buffer (5% goat serum and 0.3% Triton X-100 in phosphate buffer saline) for 30 minutes at 37°C before incubating overnight with the primary antibodies at 4°C. Secondary antibodies were incubated at 37°C for 1 hour before washing with the phosphate buffer saline. The nucleus was stained using DAPI (0.8 *μ*g/mL, Beyotime, #ZLI-9557, Shanghai, China). The tissues were then mounted onto slides and viewed under a Leica fluorescence inverted microscope (Leica Microsystems, Wetzlar, Germany).

### 2.10. Statistical Analysis

All data were presented as mean ± standard deviation (Mean ± SEM). Statistical analysis of experimental parameters was performed with independent sample *t*-test and one-way analysis of variance (ANOVA). The independent sample *t*-test is used for comparison between two groups, and the comparison of more than two groups uses analysis of variance. The Dunnett family error rates were chosen for unequal sample sizes, otherwise the LSD test was selected. Statistical analysis was performed using SPSS21.0 software (IBM, Armonk, NY, USA). The significance of the difference between groups was calculated according to Fisher's least significant difference post hoc test. Probability values (*P*) of less than 0.05 were considered to represent statistical significance.

## 3. Results

### 3.1. Intersection Protein Targets Screening and PPI Network Diagram

**Figure 1:**
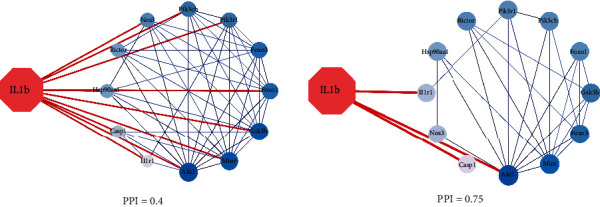
Protein-protein interaction (PPI) network. (a) PPI network of protein target (confidence ≥ 0.4), (b) PPI network of core protein target (confidence ≥ 0.75).

We constructed the PPI network interaction map of the target protein of IL-1*β* through string database analysis of the intersection protein targets of IL-1*β*, shown in Figure 1(a). The 13 core protein targets (such as AKT1, PIK3CB, CASP1, MTOR, FOXO3, and GSK3B) were obtained by setting the interaction score (confidence degree ≥ 0.75), and the core PPI network was constructed by using the core protein targets, shown in Figure 1(b).

### 3.2. GO and KEGG Enrichment Analysis

**Figure 2:**
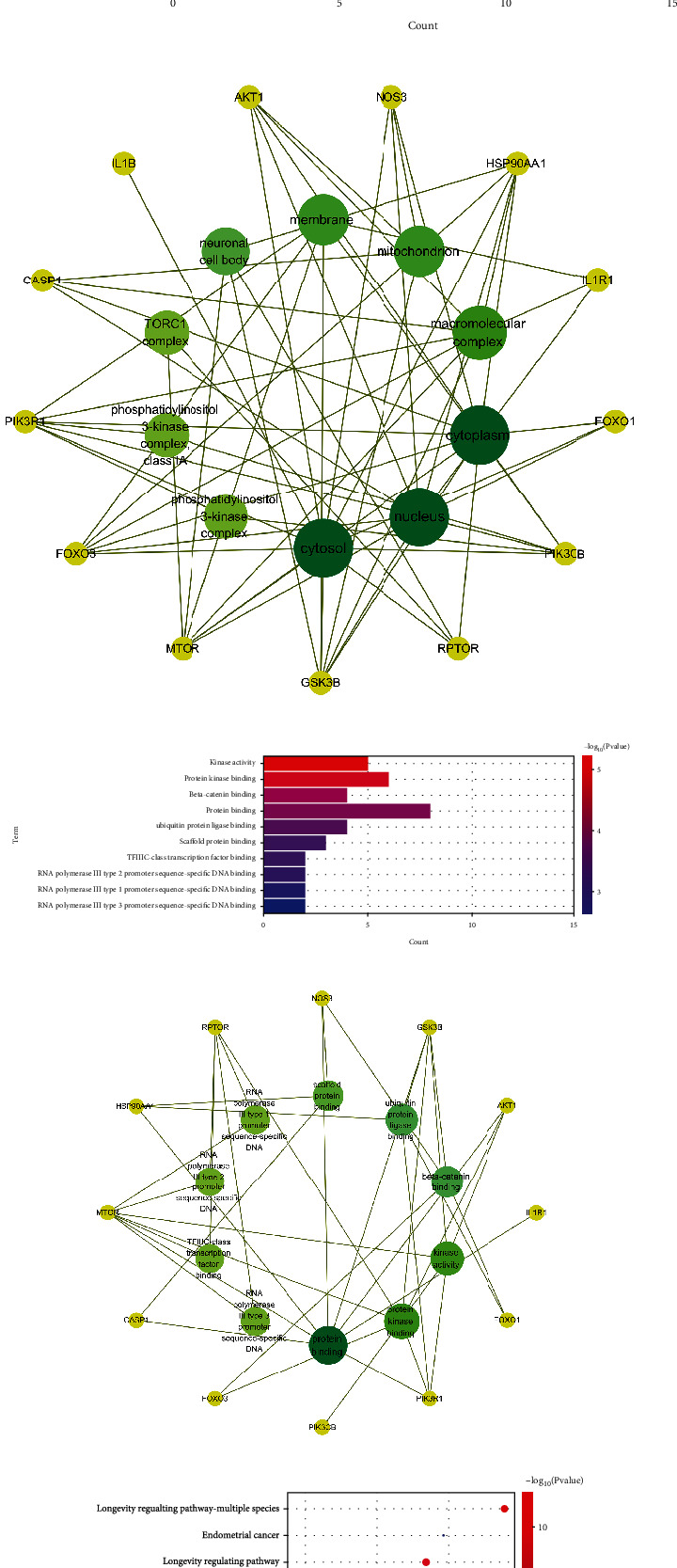
Gene Ontology (GO) and Kyoto Encyclopedia of Genes and Genomes (KEGG) analysis of related genes. (a) The top 10 terms in biological processes (BP) were greatly enriched. (b) The subnetwork displayed the top 10 BP terms and related genes. (c) The top 10 terms in molecular function (CC) were greatly enriched. (d) The subnetwork displayed the top 10 CC terms and related genes. (e) The top 10 terms in cellular components (MF) were greatly enriched. (f) The subnetwork displayed the top 10 MF terms and related genes. (g) The top 15 KEGG pathways were greatly enriched. (h) The subnetwork displayed the top 15 KEGG pathways.

The 13 core intersection gene targets were imported into the DAVID database for enrichment analysis. Under the condition of *p* < 0.05, the GO enrichment analysis yielded a total of 198 GO entries, including 150 biological process (BP) entries, 19 cellular component (CC) entries, and 29 molecular function (MF) entries. The results showed that biological processes were highly correlated with inflammation and apoptosis, mainly involving the response to xenobiotic stimulus, positive regulation of apoptotic process, and positive regulation of gene expression. Among cell components, macromolecular complex, cytosol, and nucleus are accounted for a relatively large amount. In molecular functions, kinase activity, protein kinase binding, and protein binding were relatively high, shown in Figures 2(a–f). KEGG pathway analysis yielded 110 pathways with *p* < 0.05. The results showed that the enriched pathways involve multiple pathways related to immune response and cell survival, mainly longevity regulating pathway, prostate cancer, PI3K-AKT1 signaling pathway, and other signaling pathways, shown in Figures 2(g and h).

### 3.3. Motor Function Evaluation and IL-1*β* Expression in SCC Rats

**Figure 3:**
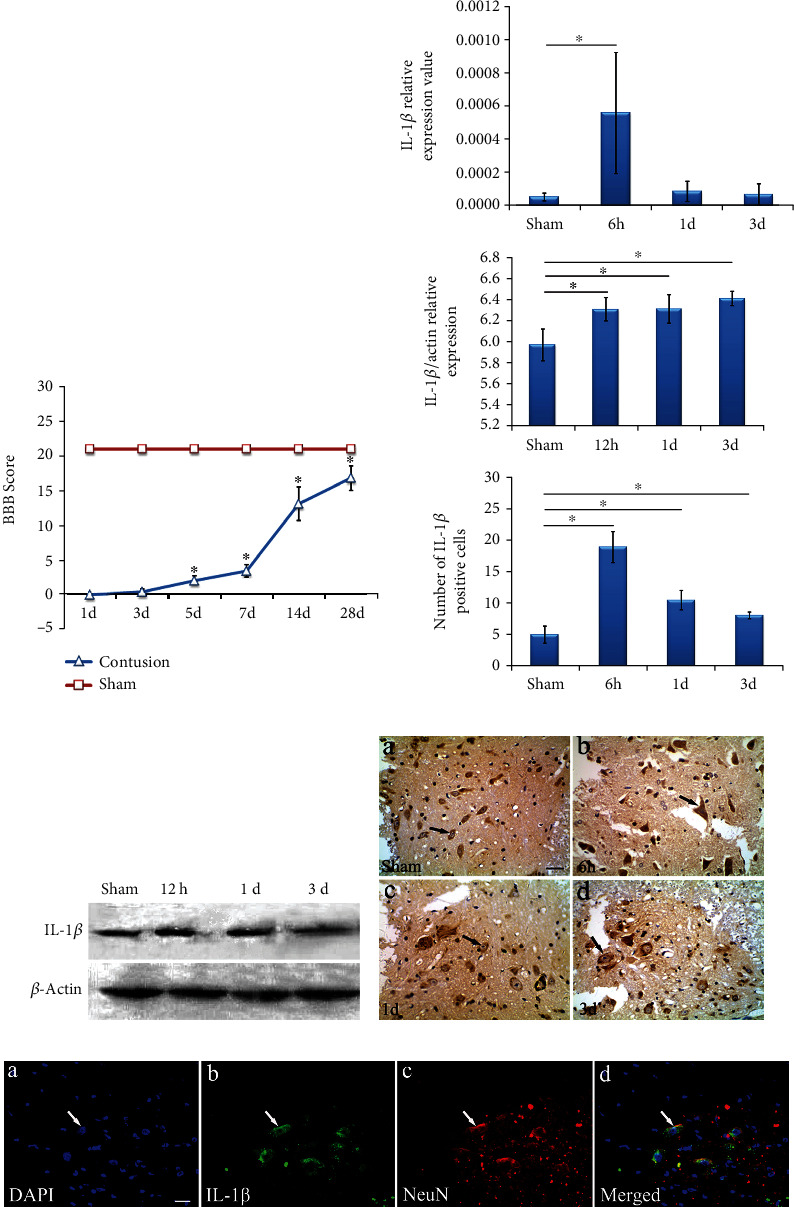
Expression of IL-1*β* at the spinal cord. (a) BBB scores in the spinal cords of contused rats on days 1, 3, 5, 7, 14, and 28. BBB scores were significantly lower and increased gradually in the contusion group compared with the sham group. (b) Expression of IL-1*β* mRNA was detected by qPCR. Data showed that IL-1*β* mRNA drastically increased and peaked at 6 hours, but there was no obvious change on days 1 and 3 when compared with the sham group. (c) IL-1*β* was detected by Western blotting, with *β*-Actin as standard value. Expression of IL-1*β* increased after spinal cord contusion when compared with the sham group. (d) Immunohistochemistry showed that IL-1*β* was located in the neurons of the anterior horn of the spinal cord. Bar = 40 × 10 *μ*m. IL-1*β* positive cells were found in the sham group and significantly upregulated at 6 hours and on days 1 and 3. (e) Immunofluorescence double staining for IL-1*β*/NeuN in neurons of the anterior horn of the spinal cord. DAPI stained blue and IL-1*β* stained green, NeuN stained red. Coexpression of NeuN with IL-1*β* within the same cell showed orange (merged). Bar = 40 × 10 *μ*m. ^∗^*p* < 0.05 when compared with the sham group.

In the 28-day study, the BBB score for the sham group was 21. In comparison, motor function of rats following SCC was significantly suppressed on day 1, but BBB scores gradually increased on days 5, 7, 14, and 28 (*p* < 0.05), shown in Figure 3(a). qPCR results showed that the expression of IL-1*β* mRNA drastically increased (*p* < 0.05) at 6 hours after SCC and returned to normal when compared with the sham group, shown in Figure 3(b). IL-1*β* protein was detected by Western blot and showed that IL-1*β* was significantly increased after SCC at 12 hours and 1 and 3 days, shown in Figure 3(c). IL-1*β* immunoreactivity was found in the spinal cord anterior horn neurons. Compared with the sham group, IL-1*β* positive cells in the SCC rats were significantly upregulated at 6 hours (*p* < 0.05). Furthermore, through immunofluorescence IL-1*β* was observed in the cytoplasm of neurons, shown in Figures 3(d and e).

### 3.4. IL-1*β* in the Recovery of Motor Function after SCC

**Figure 4:**
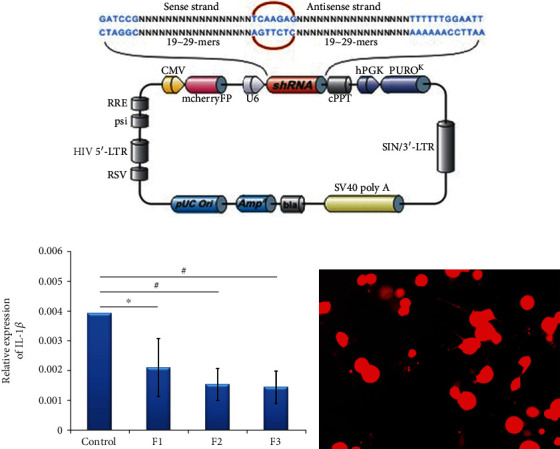
Preparation of IL-1*β*-RNAi-LV recombinants. (a) Schematic representation of IL-1*β*-RNAi-LV, in which, enhanced red fluorescent protein (mCherry FP) as the reporter gene was inserted into the plasmid. The framework also contains the antibiotic ampicillin and the plasma cloning vector pUC Ori, which were used as promoters for vector expression. (b) Screening of effective interference fragment for IL-1*β* inhibition showed that F3 exhibited the most effective interference of the three tested fragments. (c) Fluorescent image of IL-1*β*-RNAi-LV transfected into 293 T cells.

The recombinant IL-1*β*-RNAi-LV was prepared for use, shown in Figure 4(a). IL-1*β* lentiviral expression vector was prepared as follows. Three targets of IL-1*β* siRNA sequences were transfected into 293 T cells to screen out the most effective siRNA vector by real-time PCR, shown in Figure 4(b). Results showed that IL-1*β* expression ratios of F1, F2, and F3 compared with the controls were decreased, thus F3 was considered the suitable target virus and was then transfected into a primary culture of 293 T cells. Immunofluorescence was used to determine expression of the vector. Results showed that IL-1*β* expression (red) could be found in 293 T cells after transfection, shown in Figure 4(c). To construct the IL-1*β* recombinant vector, a segment of F3 was inserted into pcDNA3 plasmid and enveloped by lentivirus with routing methods.

**Figure 5:**
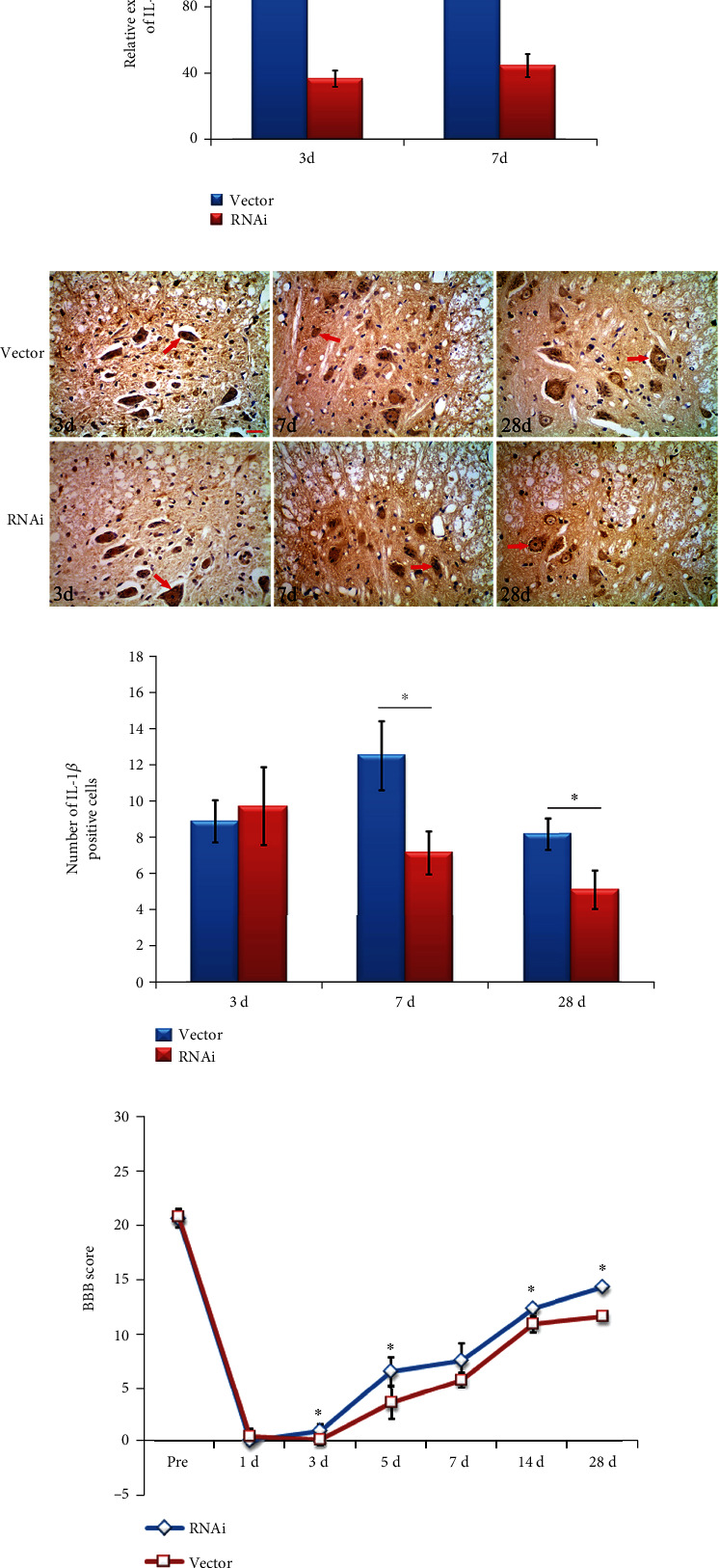
IL-1*β* in rats following spinal cord contusion. (a) Expression of IL-1*β* mRNA was successfully decreased IL-1*β*-lentiviral group. Mean values in the RNAi group were lower than in the vector group. (b) IL-1*β*-positive staining was located in the cytoplasm of neurons in the anterior horn of the spinal cord. (c) Number of IL-1*β*-positive cells in the IL-1*β*-lentiviral group were decreased on days 7 and 28. ^∗^*p* < 0.05 when compared with the vector group. (d) BBB scores in the IL-1*β*-lentiviral group were significantly higher than the vector group on days 3, 5, 14, and 28.

The IL-1*β* lentiviral vector was injected into the spinal cord of rats to verify the role of IL-1*β* in the spinal cord. qPCR was used to detect the expression of IL-1*β* in the spinal cord. Results found that the level of IL-1*β* mRNA decreased significantly when compared to the vector group (*p* < 0.05), indicating that the IL-1*β* lentiviral vector was successfully transfected on the spinal cord, shown in Figure 5(a). Meanwhile, immunohistochemistry results showed that IL-1*β* immunoreactivity was located in spinal cord anterior horn neurons and the numbers of IL-1*β* positive neurons were significantly less than the vector group on days 7 and 28 (*p* < 0.05), shown in Figure 5(c). Finally, the movement of rat hind limbs was assessed using the BBB scale. Scores in the IL-1*β*-RNAi-LV group were markedly higher than the vector group on days 3, 5, 14, and 28 following spinal cord contusion (*p* < 0.05), shown in Figure 5(d).

### 3.5. IL-1*β* in the AKT1 in PI3K/AKT1 Signaling Pathway

**Figure 6:**
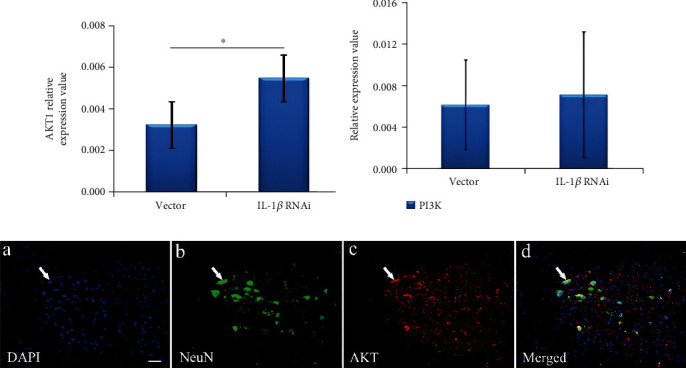
Localization and expression of AKT1 and PI3K after lentiviral interference with IL-1*β*. (a) Expression of AKT1 mRNA was detected by qPCR, which showed that expression of AKT1 mRNA was significantly increased on day 7 in the IL-1*β*-lentiviral group. (b) Expression of PI3K mRNA was detected by qPCR, which showed that expression of PI3K mRNA was not significant between the IL-1*β*-lentiviral group and vector group. (c) Immunofluorescence double staining of AKT/NeuN was showed DAPI staining blue (a), NeuN staining green (b), and AKT1 staining red (c). AKT1 was located in neurons of the anterior horn in the spinal cord (merged) and coexpressed with IL-1*β*. ^∗^*p* < 0.05 when compared to the vector group.

qPCR was used to detected the relationship between IL-1*β*, AKT1, and PI3K. Results showed that the expression of AKT1 mRNA in the IL-1*β* lentiviral group was significantly higher than in the vector group (*p* < 0.05), shown in Figure 6(a), while expression of PI3K mRNA in the IL-1*β* lentiviral group was not statistically significant compared to the vector group, shown in Figure 6(b). These results suggest that IL-1*β* might be associated with AKT1 instead of PI3K. Furthermore, immunofluorescence showed that AKT1 was located in the cytoplasm of neurons, which coexpressed with IL-1*β* in the spinal cord, shown in Figure 6(c).

## 4. Discussion

**Figure 7:**
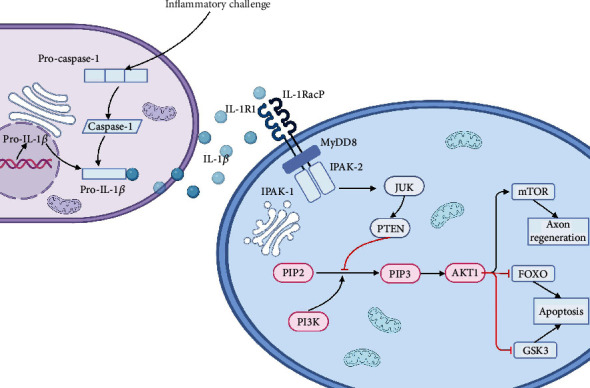


Inflammation and apoptosis of cells after spinal cord contusion are important causes of irreversible spinal cord injury, and IL-1*β* is a key inflammatory factor that contributes to the exacerbation of spinal cord contusion. However, the regulatory mechanism of IL-1*β* in spinal cord contusion is still unclear. Although bioinformatics and big data mining suggested that IL-1*β* was closely related to many key gene targets (such as AKT1 and PI3K), the simulation analysis of bioinformatics could not clarify the mutual regulatory relationship between them. Therefore, we validated the results of bioinformatics analysis by animal experiments and lentiviral interference techniques. Firstly, our experimental results suggested that increased IL-1*β* expression in spinal cord tissue after SCC hinders the recovery of motor function in spinal cord contusion. Secondly, inhibition of IL-1*β* expression by lentivirus-mediated RNAi up-regulated the expression of AKT1 in the PI3K/AKT1 signaling pathway, which was beneficial to tissue regeneration and recovery of spinal cord function after SCC. Finally, our findings suggested that IL-1*β* impeded the recovery of motor function after SCC in rats by disrupting AKT1 expression in the PI3K/AKT1 signaling pathway. And bioinformatics analysis suggested that AKT1 may affect apoptosis, regeneration, and recovery of motor function after SCC in rats by regulating the downstream FOXO, mTOR, and GSK3 signaling pathways. The summary of the mechanisms analysis of regulation of IL-1*β* on PI3K/AKT1 signaling pathway is shown in Figure 7.

### 4.1. IL-1*β* is Highly Expressed in Neuronal Cells in Acute Phase after Spinal Cord Contusion

Bioinformatics analysis suggested that IL-1*β* was involved in the activation of multiple inflammatory responses, and GO results suggested that IL-1*β* was mainly located in the cytoplasm of cells. Experimental results indicated that the BBB score was significantly lower in the spinal cord contusion group compared with the sham group. Furthermore, expression of IL-1*β* in motor neurons of the anterior horn of the spinal cord increased markedly compared with the control group at 6 hours and 1 and 3 days after SCC, and continued until day 28. Both the mRNA and protein of IL-1*β* were up-regulated in the early stage of spinal cord contusion and localized in the neuronal cytoplasm in the spinal cord contusion model, the localization of IL-1*β* in the spinal cord is in good agreement with the results of bioinformatics analysis. These results suggest that high expression of IL-1*β* in the neuronal cytoplasm has a huge impact on the acute phase of spinal cord contusion.

As an important inflammasome-related proinflammatory factor, IL-1*β* is closely related to a variety of central nervous system diseases, including spinal cord contusion, stroke, traumatic brain injury, Alzheimer's disease, etc [17]. The production of IL-1*β* in spinal cord contusion is due to nerve cell damage and proinflammatory factors by inducing microglia to produce inactive IL-1*β* precursors, and further cleavage by caspase-1 to produce effective IL-1*β*, this process is very fast [18]. Studies have demonstrated that IL-1*β* mRNA is transcribed minutes after injury, but only at the site of injury [18]. It was widely distributed in spinal cord segments at 15-45 minutes, and IL-1*β* continued to rise to the highest level within 12 hours, and then continued to decline, which was basically consistent with our finding [19]. In addition, further studies showed that IL-1*β* was first expressed in the central neurons of lesions and mainly located in microglia and astrocytes from 3 h to 12 h. Interestingly, the re-elevation of IL-1*β* around day 7 may be caused by blood-derived lymphocytes [18]. Therefore, IL-1*β* mediated early inflammation is rapid and extensive and plays an important role in the acute phase of spinal cord contusion.

### 4.2. High Expression of IL-1*β* in the Acute Phase of Spinal Cord Contusion Hinders the Recovery of Motor Function

To explore the effect of IL-1*β* on the recovery of motor function following SCC, we constructed a lentiviral vector to inhibit expression of IL-1*β*. BBB score increased significantly in the IL-1*β*-RNAi inhibitory group than in the control group. In addition, expression of IL-1*β* decreased in the SCC groups. Results suggested that inhibiting expression of IL-1*β* promotes recovery of motor function after SCC.

Interestingly, studies have found IL-1*β* to be contradictory. Early experiments have shown that IL-1*β* can induce nerve growth in vitro, and the combined effect of NT-3 significantly increases the growth rate [20] and is also required for oligodendrocytes to promote remyelination [21], so it is speculated that IL-1*β* can also promote functional recovery in vivo. However, more and more studies have demonstrated that IL-1*β* causes neuronal cell degeneration and death in CNS models [7]. IL-1*β* can also activate microglia and astrocytes to secrete inflammatory mediators leading to neurotoxicity [22, 23]. Boato et al. found that IL-1*β* increased lesion size, neuronal apoptosis and the potential to inhibit axonal growth in CNS injury in mice [9], and Hook et al. used an IL-1 receptor antagonist (IL-1ra) to promote the reduction of lesion size after spinal cord contusion [8, 24]. Furthermore, IL-1*β* knockout model (IL-1*β* KO) mice have significantly improved SCC recovery compared to wild-type animals [10]. Therefore, these results suggest that the high expression of IL-1*β* in the acute phase of spinal cord contusion hinders the functional recovery of locomotor activity in rats.

### 4.3. Upregulation of AKT1 Expression by Interfering with IL-1*β* Improves Motor Recovery in Spinal Cord Contusion

Bioinformatics results suggested that there is a regulatory relationship between IL-1*β* and AKT1. Moreover, after lentiviral interference with IL-1*β*, the expression of AKT1 increased and PI3K expression remained unchanged, and motor function was significantly restored in rats with spinal cord contusion. The experimental results showed that interfering with IL-1*β* can upregulate the expression of AKT1, thereby affecting the functional recovery of spinal cord contusion, but PI3K is not affected by IL-1*β*.

AKT1 is a serine-threonine kinase involved in the regulation of apoptosis and proliferation [25, 26]. Studies have found that AKT1 affects the recovery of spinal cord contusion by regulating downstream factors (such as mTOR, FOXO, and glycogen synthase kinase GSK3). Firstly, it was found in a spinal cord contusion model that AKT1/mTOR was involved in the regulation of neuronal autophagy and apoptosis [27], and inhibition of PTEN can promote autophagy through AKT1/mTOR [28]. Secondly, AKT1 increases GAP43 expression by inhibiting GSK3 and promotes neuronal axon regeneration [28, 29]. Finally, activated AKT1 can directly phosphorylate FOXO, resulting in the concomitant inactivation of FOXO translocation from the cytoplasm to the nucleus. AKT1 reduces apoptosis and inflammatory responses by inhibiting FOXO [30, 31].

PI3K/AKT1 is one of the major signaling pathways that have been identified for cell survival. Activation of PI3K catalyzes the conversion of PIP2 to PIP3 through stimulation of receptor tyrosine kinases and concomitant assembly of receptor PI3K complexes [32]. PIP3 acts as a second messenger to activate AKT1 and activates AKT1 through phosphorylation [33]. However, the experimental results showed that the expression of PI3K remained unchanged after lentiviral inhibition of IL-1*β*, indicating that the regulation of AKT1 by IL-1*β* may not be through the PI3K/AKT1 pathway. Study showed that IL-1*β* can activate PTEN through JNK [34, 35]. Babaev et al. reported that JNK could reduce autophagy by inhibiting AKT1 in macrophages. And after inhibiting PTEN, the inhibitory effect of JNK on AKT1 disappeared, indicating that JNK may inhibit the expression of AKT1 through PTEN [36]. PTEN acts as an inhibitor of AKT1 activation by dephosphorylating PIP3 to PIP2, thereby preventing AKT activation [37]. Therefore, the inhibitory effect of PTEN on AKT1 is weakened after interfering with IL-1*β*, thereby increasing the expression of AKT1, which can explain the reason why PI3K is not affected by IL-1*β* [38].

In conclusion, IL-1*β* may affect neuronal apoptosis and regeneration through the regulation of mTOR, FOXO, and GSK3 expression via the PTEN/AKT1 signaling pathway, thereby affecting neuronal survival and functional recovery after spinal cord contusion.

## 5. Conclusion

This project used bioinformatics to screen out IL-1*β* and AKT1 that affect neuron survival after spinal cord contusion. In order to verify and further explore their interaction and regulatory mechanism after spinal cord contusion, we established a model of blunt spinal cord contusion in adult female rats. Experiment demonstrated that inhibition of IL-1*β* mediated by lentivirus contributed to the recovery of motor function in SCC rats. IL-1*β* may affect apoptosis and regeneration by inhibiting AKT1 expression in the PI3K/AKT1 signaling pathway regulating the downstream FOXO, mTOR, and GSK3 signaling pathways, thereby impeding the recovery of motor function after SCC in rats.

## Figures and Tables

**Table 1:** Selection of primers for qPCR experiments.

Gene name	Primer sequence (sense)	Primer sequence (antisense)
*β*-Actin	GAAGATCAAGATCATTGCTCCT	TACTCCTGCTTGCTGATCCA
Il-1*β*	GAGCTGAAAGCTCTCCACCT	TTCCATCTTCTTCTTTGGGT
AKT1	GAGAACCTCATGCTGGACAAG	GTCGTTGTCCTCCAGCACCT
PI3K	TATGCCTGCTCTGTAGTGGT	TAGTGACATTGAGGGAGT

## Data Availability

A preprint has been previously published [June 2020]. The data that support the findings of this study are available in ResearchGate at [DOI: 10.21203/rs.3.rs-33767/v1], reference number: [37].

## Abbreviations

SCC: Spinal cord contusion
IL-1*β*: Interleukin-1 beta
AKT: Protein kinase B
PI3K: Phosphatidylinositol-3-kinase
BBB: Basso Beattie Bresnahan
RNAi: RNA interfering
SCC: Spinal cord contusion
CNS: Central nervous system
IL-1ra: IL-1 receptor antagonist
SD: Sprague-Dawley
IP: Intraperitoneal.

## Supplementary Materials

Supplementary Materials Bioinformatics analysis data has been uploaded as supplementary file.
